# TRIzol and Alu qPCR-based quantification of metastatic seeding within the skeleton

**DOI:** 10.1038/srep12635

**Published:** 2015-08-14

**Authors:** J. Preston Campbell, P. Mulcrone, S. K. Masood, M. Karolak, A. Merkel, K. Hebron, A. Zijlstra, J. Sterling, F. Elefteriou

**Affiliations:** 1 Department of Pharmacology, Vanderbilt University, Nashville, Tennessee, United States of America; 2 Department of Pathology, Microbiology, and Immunology, Vanderbilt University, Nashville, Tennessee, United States of America; 3 Department of Veterans Affairs (VISN 9), Nashville, Tennessee, United States of America; 4 Vanderbilt Center for Bone Biology, Vanderbilt University, Nashville, Tennessee, United States of America

## Abstract

Current methods for detecting disseminated tumor cells in the skeleton are limited by expense and technical complexity. We describe a simple and inexpensive method to quantify, with single cell sensitivity, human metastatic cancer in the mouse skeleton, concurrently with host gene expression, using TRIzol-based DNA/RNA extraction and Alu sequence qPCR amplification. This approach enables precise quantification of tumor cells and corresponding host gene expression during metastatic colonization in xenograft models.

Numerous imaging technologies exist for assessing skeletal tumor burden in preclinical cancer metastasis models, but all require a well-established tumor to generate sufficient signal. Smaller numbers of metastatic cells can be detected with flow cytometry, but this approach is costly, requires marrow flushing, and is prone to high inter-sample variability. Understanding the mechanisms that drive early skeletal establishment of metastatic cancer cells requires more sensitive and quantitative methods than those currently in common use. One such method relies on the presence of *Alu* tandem repeats in the human genome, which was first documented three decades ago^1^. These short, interspersed fragments (SINEs) are ~300 bp retrotransposons, repeated more than 10^6^ times per genome, and exclusively in higher primates. The vast number of these sequences has allowed them to be used as a very robust detection tool in anthropology and forensics for identifying human elements in ancient bone samples^2^ and remains^3^. The potential for using real-time qPCR amplification of human repeats to accurately quantify small numbers of cells *in vivo* in scientific investigations has been known for more than 20 years^4^, but this technique has had limited value due to high background signals from human contamination and the challenges of consistent DNA extraction across different tissue types^5^.

Technical difficulties inherent in tissue extraction of DNA are compounded in bone. The entrapment of DNA in the calcified organic material of bone and shells is beneficial for archaeologists and paleontologists, as these chemical interactions can stabilize and preserve DNA for centuries^6^,^7^. However, these same interactions make the process of extracting DNA from bone difficult, as it binds with hydroxyapatite^8^,^9^ and matrix proteins such as collagen^10^. Our objectives were to improve upon existing methods of nucleic acid extraction from bone and to utilize genomic human *Alu* repeats in xenograft models to establish a fast, reliable, inexpensive and highly-sensitive technique for earlier quantification of bone metastatic human tumor cells within skeletal tissues. Specifically, we set out to find a method which would allow researchers to easily acquire DNA and RNA from the same sample of bone tissue and then use these samples to accurately quantify disseminated tumor cells and corresponding host gene expression in order to determine which stromal factors contribute to metastatic establishment.

Several established nucleic acid extraction techniques were systematically evaluated and we found that a modification of the TRIzol^TM^ protocol using a back extraction buffer (TRIzol-BEB method) allowed for the most efficient extraction of DNA from bones. Yields were superior to those obtained with a commercial kit (DNeasy kit, Qiagen) and another commonly-used protocol based on proteinase K digestion (Fig. 1a,b). It is important to note here that RNA expression data *and* DNA can be extracted from the same sample with the TRIzol-BEB method, while the other techniques yield only genomic information to be quantified. Other methods of DNA extraction from bone, which did not consistently yield serviceable DNA, included using EDTA decalcification, NaOH and boiling, or other TRIzol modifications recommended by the manufacturer (data not shown). For TRIzol-BEB DNA extraction, frozen samples were pulverized using a carbon steel mortar and pestle, previously cooled in liquid nitrogen. By keeping the surface of the pestle between -100 and 0^ °^C, we were able to exploit the Leidenfrost effect, which slows the evaporation time of liquid nitrogen and allows for nucleation of the suspended sample powder on the collection tool, thereby significantly reducing contamination and sample loss. Powdered samples were then resuspended in TRIzol and thoroughly vortexed before proceeding with extraction and precipitation. The range of RNA yield was 0.23–1.24 μg per mg of starting tissue, (x̄ = 0.63 μg/mg of tissue). Cross-contamination was not detectable in the RNA or DNA, which were extracted from the same sample, as shown by gel electrophoresis (Fig. 1c).

In addition to problems from RNA-DNA cross-contamination, efficient PCR of nucleic acid extracts from bone can be inhibited by excess Ca^2+^ ions and protein impurities, which are not detectable on agarose gel analyses, but RNA and DNA extracted with the TriZOL-BEB method showed no evidence of PCR inhibition, as shown by: A260/A280 ratios between 1.71 and 1.97 (x ¯= 1.85 for DNA) and 1.54 and 2.10 (x̄ = 1.89 for RNA) (Supplementary Fig. 1a–d), and linear qPCR amplification values of bone mouse *β-actin* in serial dilutions (Fig. 1d), with starting DNA concentrations as high as 200 ng. *Alu* detection of 10^3^ human MDA-MB-231 breast cancer cells in 20ul PBS pipetted directly,“spiked”, into bone samples (~75 mg of tissue), by qPCR showed no evidence of either degradation or inhibitors in the extracted samples, as shown by slope and R^2^ of Ct Alu plot (Fig. 1e), even at 10-fold DNA concentration. In both RNA and DNA extraction, inhibitor carryover at the precipitation step could be greatly reduced by using isopropanol extraction rather than ethanol and salt^11^.

Though qPCR of primate *Alu* repeats has been used routinely in forensics and anthropology, attempts at using *Alu* qPCR to quantify human tumor cells in xenograft models have been hampered by extraction difficulties and background signal from human DNA contamination. Genomic DNA extracted using our modified TRIzol-BEB protocol was used for *Alu* qPCR and gave accurate linear quantification of 10^1^ to 10^6^ human cell numbers from serial dilutions of MDA-MB-231 cells spiked into 10^6^ murine bone marrow cells (Fig. 2a). Accurate cell number counts were also seen when *Alu* qPCR was used to count MDA-MB-231 cells that were spiked directly into entire pulverized mouse femora (Fig. 2b), demonstrating that the combined methods of TRIzol-BEB extraction and *Alu* qPCR provide a sensitive method to quantify disseminated human metastatic cells in bone tissue.

Although we were able to detect the presence of 1 human cell within 10^6^ mouse bone cells (Fig. 2a) and 10 human cells per bone (Fig. 2b), the accuracy of these data is limited by the inherent imprecision of using serial dilutions to isolate a single cell. To overcome this technical limitation and determine the maximum sensitivity of the method, we used fluorescence assisted cell sorting (FACS) to single-cell sort between 1 and 64 GFP-expressing MDA-MB-231 cells into 10 μL of media, which was added directly to an entire crushed mouse humerus, containing approximately 10^7^ bone cells. After using TRIzol-BEB extraction to obtain gDNA from these samples and performing *Alu* qPCR, we were able to distinguish the difference in signal between 1 and 2 cells (Fig. 2c), both of which were above background.

Among currently used techniques for quantifying cell numbers, flow cytometry at present has the most sensitivity, theoretically capable of detecting a single cell within any population of cells. We compared its sensitivity to the TRIzol-BEB-Alu qPCR technique for quantifying cell numbers *ex vivo,* by serial dilution of human MDA-MB-231 cells into bone marrow cells flushed from mouse femora. Quantification of MDA-MB-231 cells by flow cytometry, though very precise, estimated the cell counts at 1/3 of the actual number (Fig. 2d,e). Furthermore, the background signal in the flushed marrow limited the sensitivity to 1 in 10^5^ cells, which is less than what was achieved with qPCR of Alu sequences (Fig. 2c). These results demonstrate that qPCR of *Alu* repeats is more accurate, precise, and sensitive than FACS for the detection of human cancer cells in mouse bones. In addition to being more expensive, flow cytometric methods are also technically hampered by the requisite of flushing the marrow from the bone in order to obtain cells. Though marrow extraction techniques have been in the literature since 1945, these methods do not allow an exhaustive removal of cells from the bone, which is needed to consistently locate small tumor foci by FACS or histology. Metastatic tumor cells in bone typically colonize near metaphyses rather than in the diaphyseal marrow and most of these cells remain undisturbed after marrow flushing (Supplementary Fig. 2a–d), thereby preventing subsequent FACS analysis of the tumor cells detection.

In the commonly-used preclinical models of bone metastasis, it has been speculated that very few of the metastatic cells survive, and that most die over the course of the first week, resulting in a trough of bioluminescent signal during this first week. Attempts have been made with fluorescence or bioluminescence approaches to quantify the events in the early hours or days of metastatic establishment, but these techniques are limited by low reporter gene expression seen in metabolically quiescent cells^12^, limited depth of penetration and scattering of signal^13^, multiple layers of tissue between deep metastatic cells and the detector, and by the relative opacity of dense tissues like bone. Given these limitations, we applied the Alu qPCR technique to reveal the fate of bone metastatic cells in the intracardiac mouse metastasis model, and compared the results with eGFP fluorescence data and eGFP RNA expression from the same samples. According to Alu qPCR, 10–100 cells arrested in any one long bone at 3 hours post intracardiac injection, and cell number increased steadily until mice were sacrificed at 14 days (Fig. 3a). In contrast to the increasing number of cells quantified by Alu qPCR, eGFP qPCR of MDA-MB-231 cells that express eGFP driven by the CMV promoter (Supplementary Fig. 3a) showed a decrease from d1 to d7 that would suggest a reduction in cell number in the tissues. When *in vitro eGFP* and *Alu* qPCR signals were compared from MDA-MB-231 cells alone, the slopes were not significantly different (Supplementary Fig. 3a–b) which excludes the possibility of differences in qPCR efficiency between eGFP cDNA and Alu genomic DNA. The discrepancy between *in vivo* and *in vitro* Alu DNA vs eGFP RNA signal may be due to metabolic changes in tumor cells, which are known to affect expression of reporter genes, even when under the control of a constitutive promoter^14^. Using fluorescence imaging, we did not observe an *ex vivo* signal in bone until 9 days after injection, which corresponded to >1000 cells (Fig. 3b), consistent with previous findings regarding the sensitivity of spectral imaging *in vivo*^15^. Furthermore, many large tumor foci were rendered undetectable by *ex vivo* fluorescence; by simply adjusting the positional angle of the bone during imaging and fluorescence, the resulting data- used to quantify tumor burden- were highly variable (data not shown). Interestingly, the number of bones with detectable metastases (more than 10 Alu + cells) did not increase over two weeks, though detectable metastases by *ex vivo* fluorescence appeared to increase (Supplementary Fig 2f). This observation further supports the advantage of Alu detection to precisely quantify the number of metastatic tumor cells in the skeleton (and other tissues) at the earliest stage of metastasis, and reinforces the recent observation that circulating tumor cell clusters may have higher metastatic potential than isolated tumor cells^16^.

We next considered that possible artifacts, such as clonal expansion of cells with low eGFP expression, decrease reporter expression in less metabolically active foci, or that hypoxic conditions in the bone metastatic site could attenuate the GFP signal and thus decrease the sensitivity of fluorescent detection methods. In order to control for these variables, we intratibially injected between 10^1^ and 10^5^ MDA-MB-231 cells which expressed both luciferase and GFP. We quantified tumor signal within 1 hour using luminescence and fluorescence, both *in vivo* and *ex vivo,* before extracting DNA and performing *Alu* qPCR. We used this short time course to minimize large scale changes in eGFP RNA that could affect the sensitivity of imaging modalities. Neither fluorescence nor luminescence imaging modalities could *consistently* detect <1000 tumor cells *in vivo*, and though both methods were sensitive for detection *ex vivo*, they could not accurately quantify absolute or relative numbers of cancer cells within bone (Fig 3c,d). *Alu* qPCR, however, was able to both detect and accurately quantify tumor cells from within the bone of all the samples (Fig. 3e).

We previously reported that chronic stress-induced expression of RANKL by osteoblasts mediates an increase in MDA-231VU metastasis to the skeleton^17^, as measured at end point (28 days post inoculation) by *ex vivo* GFP fluorescence imaging. In order to determine if the same neuroendocrine stimuli could cause quantifiable changes in tumor cell number at early post-inoculation time points, we subjected mice to chronic immobilization stress (CIS) for 10 days prior to intracardiac injection of tumor cells. Without any further treatment, we were able to measure a significant increase in the number of metastatic cells 5 days post-inoculation in the CIS group compared to controls (Supplementary Fig. 4a–b), confirming that *Alu* qPCR can be used to quantify changes in tumor cell number at the earliest stages of metastatic establishment.

The TRIzol-BEB extraction and *Alu* qPCR methods discussed herein is both more accurate and precise than existing imaging modalities for measuring absolute numbers of metastatic tumor cells in skeletal tissues, while allowing gene expression studies from the same bone biopsies. In addition to high sensitivity, the quantification of metastasis by *Alu* qPCR allows one to quantify disseminated tumor without requiring stable transfection for imaging, which can drastically change the phenotype of the tumor cells. This advantage is critical in patient derived xenograft studies where minimal manipulation of the tumor is desired in order to maintain patient phenotype and more accurately predict patient outcomes. Additionally, *Alu* qPCR relies on amplification of a robust and stable genomic signal, allowing a more sensitive, consistent and accurate cell number quantification than the inherently variable mRNA expression data of reporter genes. Importantly, this method allows for simultaneous quantification of gene expression from host mRNA and precise cell number from tumor DNA. We validated the Alu qPCR method with the two most widely used *in vivo* models of skeletal metastasis, which allowed us to precisely control both the number of cancer cells and time that samples were collected. Further studies with orthotopic xenograft models and with other cancer cell types than breast cancer will need to be performed in order to validate the combined techniques’ utility in determining the time to metastasis and the number of disseminated cells in each organ. An obvious limitation of this method is that information about the specific location of disseminated metastatic cells within the tissue is lost during pulverization. However, when combined with imaging modalities, this technique should prove a powerful tool in the study of host stromal influences on the early phases of metastatic establishment in xenograft models.

## Methods

### TRIzol DNA and RNA extraction

1 mL of TRIzol reagent was added to each powdered tissue sample (30-90 mg fresh tissue weight) on ice, followed by 10–30 sec of vortexing, to ensure that any clumps of tissue were dispersed. Samples were incubated at RT for 5 min to allow for lysis and disruption of cells. To each tube 200 μL of chloroform was added, followed by 30 sec of shaking/inversion and a 5 min incubation at RT. Samples were then centrifuged at 16000 × g for 10 min at 4 °C to separate RNA into aqueous phase. 350 μL of aqueous phase were carefully transferred to another microcentrifuge tube, and the RNA was precipitated with 500 μL of isopropanol at RT for 5 min. Samples were centrifuged at 16000 × g for 10 min at 4 °C to pellet RNA. Supernatant was removed and the pellet was washed with 1 mL of 70% EtOH, followed by a brief centrifugation at 5000 × g for 5 min. The supernatant was carefully removed and samples resuspended in 40-100 μL of DEPC-treated water, then incubated at 65 °C for 30 min to dissolve the RNA.

After RNA extraction, the remaining aqueous layer from each sample was very carefully removed. Brief centrifugation for 5 min at 16000 × g was necessary to separate phases if interphase disruption had occurred. To each sample, 400 μL Back Extraction Buffer (BEB), which is an aqueous solution of 4 M guanididium thiocyanate, 50 nM sodium citrate and 1 M Tris, was added. After 10–30 sec of vortexing, the samples were centrifuged at 16000 × g for 15 min at RT. The aqueous phase was transferred to a new tube and DNA precipitated with 400 μL of isopropanol for 5 min at RT.

### DNA purification

DNeasy Blood & Tissue Kit from QIAGEN was used according to the manufacturer’s instructions. Briefly, bone samples were snap frozen in liquid nitrogen, then pulverized using a mortar and pestle, and up to 100 mg of sample were placed into a 2 mL microcentrifuge tube in order to begin the purification. The steps described by the kit involve lysing of samples with a proteinase K-containing buffer, binding the DNA to the DNeasy Membrane of the DNeasy mini spin columns during centrifugation, 2 wash steps to remove impurities and enzyme inhibitors in the samples, and elution of the desired DNA in TE buffer or water.

Proteinase K method: samples were snap frozen in liquid nitrogen, then pulverized with a mortar & pestle and placed into a 2 mL microcentrifuge tube. 1 mL of Proteinase K lysis buffer (10 nM Tris, 100 mM NaCl, 10 nM EDTA, 0.5% SDS, 400 μg/mL Proteinase K) was added to each bone sample and then mixed by vortexing and pipetting. Samples were incubated for 2 hr at 60˚C, with intermittent mixing before debris were precipitated with excess NaCl (6 M). After centrifugation, supernatant was removed and DNA precipitated with isopropanol. Each pellet was washed with 70% EtOH and resuspended in TE or water.

Cell number data was approximated using a standard curve established by adding log-fold dilutions of human MDA-231 cells to whole murine bones *ex vivo*.

### QPCR

Real-time PCR was performed using TaqMan gene expression assays or SYBR Green on a BioRad CFX96 Real Time System. Taqman probes/primers were from Applied Biosystems. For RNA experiments, cDNA was generated using the High Capacity Reverse Transcriptase Kit (Applied Biosystems #438814). Results were analyzed using standard curve quantification or ddCt methods. The following primers and conditions were used: Human Alu, Fw: YB8-ALU-S68 5′-GTCAGGAGATCGAGACCATCCT-3′, Rev: YB8-ALU-AS244 were 5′-AGTGGCGCAATCTCGGC-3′,  Probe: YB8-ALU-167 5′-6-FAM-AGCTACTCGGGAGGCTGAGGCAGGA-TAMRA-3′. Mouse β-actin (Mm00607939_s1) was used as an endogenous control to normalize each sample and gene. PCR reactions were performed in a 10 μl volume using 0.5 μl TaqMan probe, 100 ng cDNA template, 5 μl TaqMan Gene Expression Master Mix (Applied Biosystems, Foster City, CA), and 2.5 μl DNase/RNase molecular grade water (Ambion, Austin, TX).

All TaqMan assays were performed using the following PCR conditions: denaturation at 95 °C for 15 min followed by 40 cycles of denaturing at 95 °C for 10 sec, and annealing at 60 °C for 1 min. Reactions were run in duplicate.

All SYBR Green primer pairs were validated for specificity using a Tm gradient protocol prior to the experiments performed in this manuscript. eGFP expression was assessed from RNA that was isolated from hindlimb bones of athymic nude fox3p^nu/nu^ female mice aged 4–6 weeks, which contained a certain spiked number of GFP-transduced MDA-231VU human breast cancer cells. RNA was converted to cDNA using the High Capacity cDNA Kit from Life Technologies as per the manufacturer’s instructions, and we utilized SYBR Green technology to measure gene expression. A serial dilution of MDA-231VUs was used to create a standard curve for eGFP expression. The expression of mouse HPRT, a common housekeeping gene, was also measured to assess the quality of the samples. Each reaction was performed in triplicate, and each sample was plated in technical triplicates. Using the Ct value of the y-intercept of the standard curve as a representation of 1 cancer cell, we were able to correlate the number of cancer cells the eGFP qRT-PCR yielded to the number of cells spiked into the mouse bones.

### Animal Models

All experiments using live mice were performed in accordance with the Guidelines and Regulations for the Care and Use of Laboratory Animals in AAALAC-accredited facilities, and were approved by the Institutional Animal Care and Use Committee at Vanderbilt University Medical Center. Mice were group housed in plastic cages (n = 5/cage) under standard laboratory conditions with a 12-h dark, 12-h light cycle, a constant temperature of 20 °C, and humidity of 48%. Mice were fed a standard rodent diet (Pharma Serv, Purina Rodent Laboratory Chow 5001; Framingham, MA). Nude mice were housed in sterile conditions and fed autoclaved standard chow.

### Intracardiac MDA-MB-231 Model

MDA-231VU cells were selected by serial *in vivo* passaging of GFP-expressing MDA-MB-231 cells. Briefly, cells were injected via intracardiac method, and GFP-positive cells were harvested from the long bones. The highest 10% of GFP-expressing cells were sorted via FACS.

MDA-231VU cells were cultured in 10% FBS DMEM with 1% penicillin/streptomycin. Cells were trypsinized at 70–90% confluence, rinsed and re-suspended in cold PBS at 10^6^ cells/mL. Athymic nude fox3p^nu/nu^ female mice aged 4–6 weeks were anesthetized and injected in the left cardiac ventricle with 100 μL of cell suspension (10^5^ MDA-231VU cells).

### Chronic Immobilization Stress (CIS)

CIS was carried out by placing fox3p^nu/nu^ in 50 mL laboratory conical tubes, perforated for adequate air supply, 2 hours daily , for 10 days until the time of intracardiac injection.

### Intratibial Injections of MDA-231VU Cells

MDA-231VU cells were cultured as previously stated. Cells were trypsinized at 70–90% confluence, rinsed, and re-suspended in cold PBS at 10^7^ cells/mL. Athymic nude fox3p^nu/nu^ female mice aged 4–6 weeks were anesthetized using isoflurane, and the mice were injected in the tibias at the proximal epiphysis with 10 μL of cell suspension (ranging from 0–10^5^ MDA-231VU cells). Mice we sacrificed after a certain number of days, and whole hindlimb DNA was harvested in order to perform Alu and β-actin qPCR.

### Imaging

Fluorescence data were obtained with the Maestro™ *in-vivo* fluorescence imaging system (Cambridge Research & Instrumentation) using 480 nm excitation and 515 emission filters to discriminate eGFP. Luminescence data were acquired with the IVIS 200® (Perkin Elmer) imaging platform.

### FACS

BMSCs and MDA-231VU cells were trypsinized and counted. MDA-231VU cells were spiked into 3e6 mouse BMSCs. Cells were discriminated based on cell size and granularity using forward and side-scatter analysis. Nonviable cells were gated out of further analysis. GFP gating was based on MDA-231VU run alone without BMSCs as a positive control and BMSCs run alone without MDA-231VU as a negative control. GFP analyses were conducted on a BD LSRII flow cytometer (Franklin Lakes, New Jersey, USA).

For cell-sorting experiments, MDA-231VU cells were trypsinized and sorted into 10 μL of media using a BD FACS Aria III cell sorter. Cells were discriminated based on cell size and granularity using forward and side-scatter analysis. Nonviable cells were gated out of further analysis.

## Additional Information

**How to cite this article**: Campbell, J.P. *et al.* TRIzol and Alu qPCR-based quantification of metastatic seeding within the skeleton. *Sci. Rep.* **5**, 12635; doi: 10.1038/srep12635 (2015).

## Supplementary Material

Supplementary Information

## Figures and Tables

**Figure 1:**
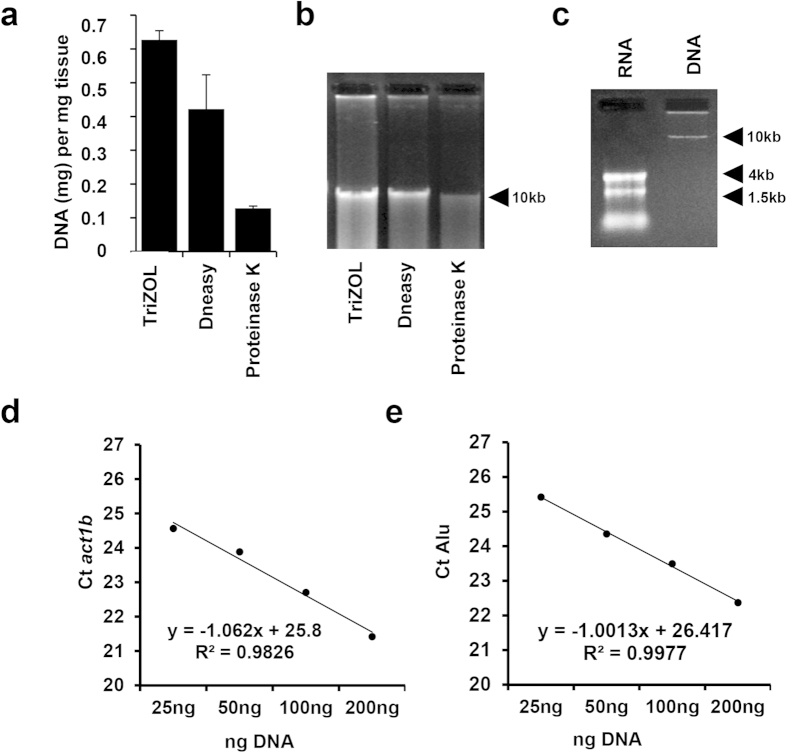
PCR-grade quality DNA and RNA from TRIzol-BEB extraction. (**a**) The concentration of DNA samples from mouse bones using 3 different protocols was determined by measuring UV absorbance (**b**) Electrophoresis of DNA extracted from mouse tibiae using different protocols on a 1% (wt/vol) agarose gel in 0.5x TAE running buffer (2.0 μg of DNA loaded in each lane). (**c**) RNA and DNA extracted with TriZOL-BEB from the same bone sample were run on a denaturing gel, showing no evidence of cross contamination in the samples (d) TriZOL-BEB method allows for extraction of DNA and RNA from bone tissues. Alu qPCR of samples with 10^3^ MDA-231 cells spiked into a mouse tibia (**d**) with corresponding mouse *b-actin* expression (**e**) using up to 200 ng DNA extracted from bone tissue, (n = 3).

**Figure 2:**
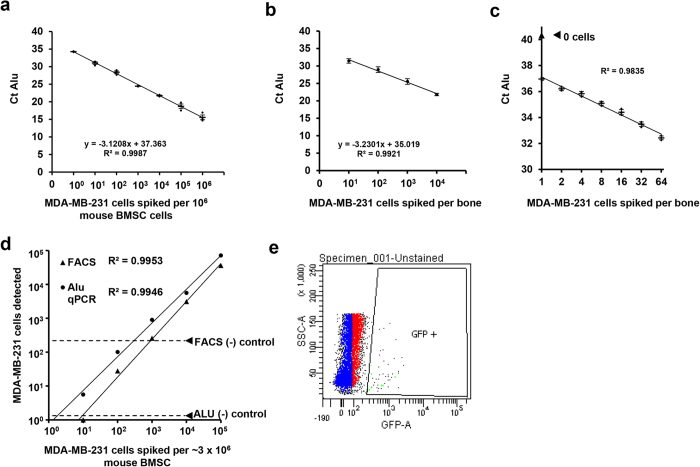
Alu PCR is a sensitive technique for detecting xenograft cells within the bone. Correlation of ct of Alu with number of human MDA-MB-231 cells spiked into murine BMSC (**a**) and whole mouse femora (**b**). (**c**) Cell number could be quantified by Alu qPCR from low numbers of MDA-MB-231GFP sorted into entire mouse humerii (). Comparison of sensitivity of FACS with Alu qPCR (**d**) in detecting MDA-MB-231GFP cells spiked into 10^6^ mouse BMSCs. Dashed lines are placed at the level of background signal from negative controls (no human cells) using the gating for eGFP shown in (**e**), n = 3.

**Figure 3:**
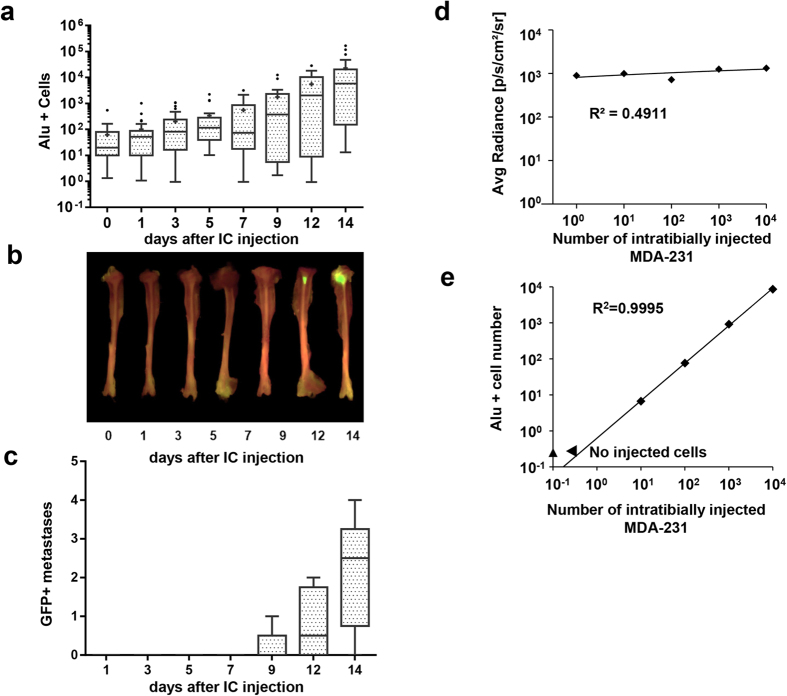
Alu PCR accurately quantifies tumor cell establishment in the bone. Representative *ex vivo* imaging of tibiae at different time points after intracardiac injection of MDA-231 tumor cells (**a**) and quantification of GFP+ bone tumors per mouse (**c**) (n = 5)with representative GFP images (**b**). BLI of tibiae injected with known numbers of MDA-231 cells expressing Luciferase (**d**) and corresponding Alu qPCR from the same bones (**e**), n = 2.
