# Supplementary material for: TRIzol and Alu qPCR-based quantification of metastatic seeding within the skeleton
Source: Sci Rep. 2015 Aug 14;5:12635. doi: 10.1038/srep12635 (PMC4536516; doi:10.1038/srep12635)
Supplement: Supplementary Information [file srep12635-s1.pdf]

TRIzol and Alu qPCR-based quantification of metastatic seeding within the skeleton

J. Preston Campbell[1,4*], P. Mulcrone[1,4], S.K. Masood[4], M. Karolak[1,4], A. Merkel[3,4], K. Hebron[2,4], A. Zijlstra[2,4], J. Sterling[3,4], F. Elefteriou[1,4]

[1]Department of Pharmacology, Vanderbilt University, Nashville, Tennessee, United States of America

[2]Department of Pathology, Microbiology, and Immunology, Vanderbilt University, Nashville, Tennessee, United States of America

[3]Department of Veterans Affairs (VISN 9), Nashville, Tennessee, United States of America

[4]Vanderbilt Center for Bone Biology, Vanderbilt University, Nashville, Tennessee, United States of America

*Correspondence to: Preston.campbell@vanderbilt.edu

Patrick.l.mulcrone@vanderbilt.edu

Sameena.k.masood@vanderbilt.edu

Alyssa.r.merkel@vanderbilt.edu

Katie.hebron@vanderbilt.edu

Andries.zijlstra@vanderbilt.edu

Julie.sterling@vanderbilt.edu

Florent.elefteriou@vanderbilt.edu

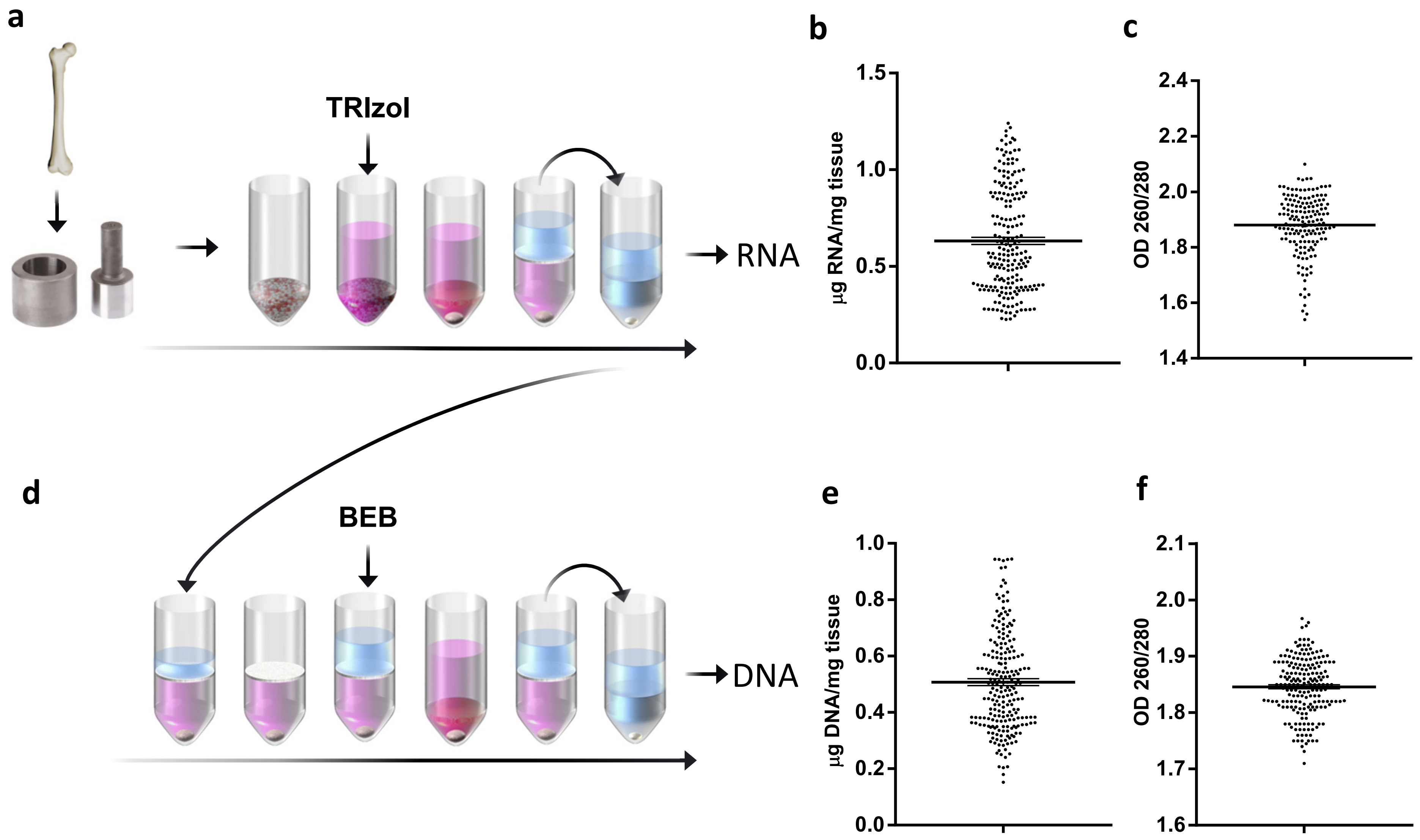

Supplementary Figure 1 | TriZOL-BEB-extraction overview a) Frozen tissue was pulverized, then TriZOL was added to each sample and immediately vortexed. After chloroform extraction, RNA was precipitated with Isoproranol, washed, and resuspended. Average RNA yield and 260/280 from mouse bones (a, b) n=200. Following RNA extraction, residual aqueous layer was removed, BEB was added followed by at least 30s of vortexing of each sample (d) . Aqueous layer was removed and sample DNA precipitated with isopropanol, washed, and resuspended. Average DNA yield and OD 260/280 after TriZOL/BEB method, n=200. Images created by JPC.

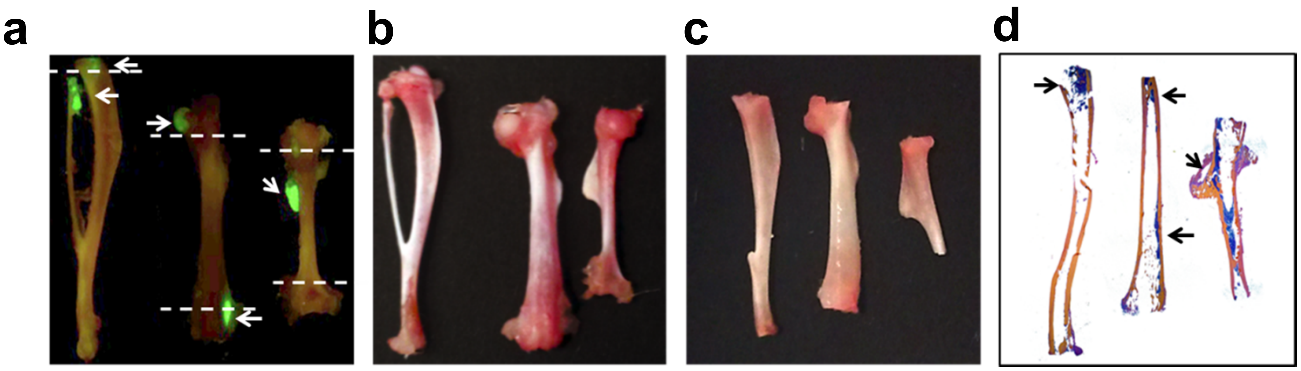

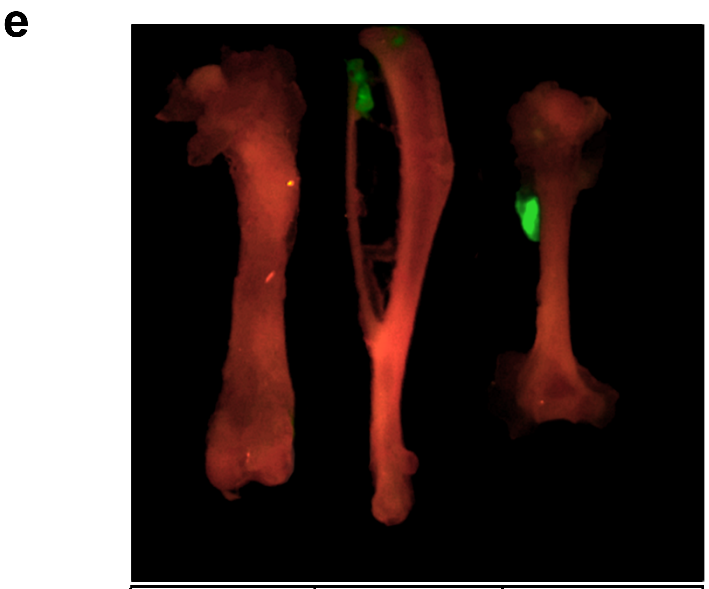

	femur	tibia	humerus
Cell # (ALU+)	3.7 x 10^4	1.8 x 10^4	5.2 x 10^3
GFP (arbitrary units)	no signal	1.6 x 10^4	4 x 10^4

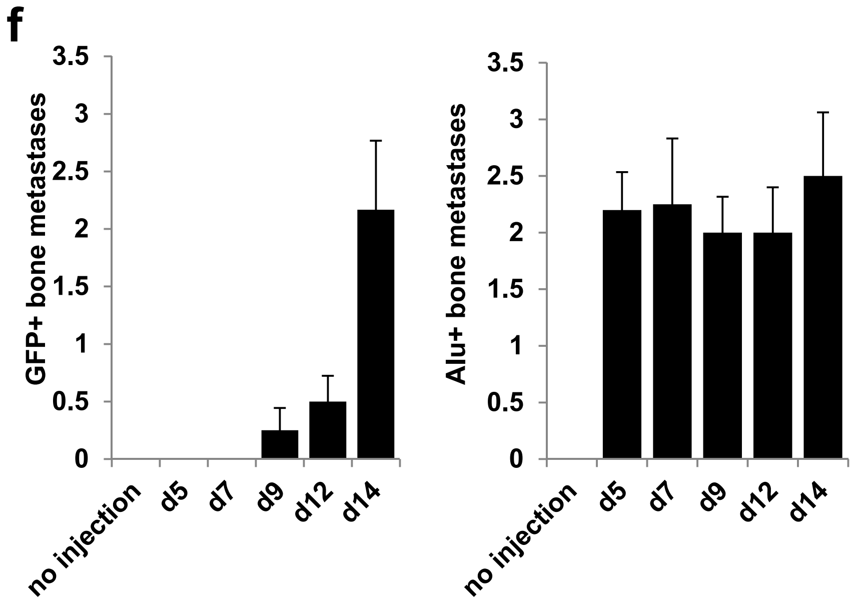

Supplementary Figure 2 – Early stage metastasis is detectable with Alu qPCR but not fluorsecence imaging. (a) Fluorescence ex vivo imaging of mouse bones with GFP+ tumors (arrows). Bones have been cleaned of excess tissue **(b)** have visible red marrow. Image of bones after ends have been cut and the marrow removed by flushing and/or centrifugation techniques **(c)**. After fixation and staining, large amounts of cells and tumor remain in the bone **(d).** Comparison of cell number quantification by Alu with fluorescent signal in the same bones **(e)** showing great variability in fluorescence intensity depending on depth of tumor and angle of bone. **(f)** Comparison of number of bone metastases detected with GFP vs Alu qPCR(n=5).

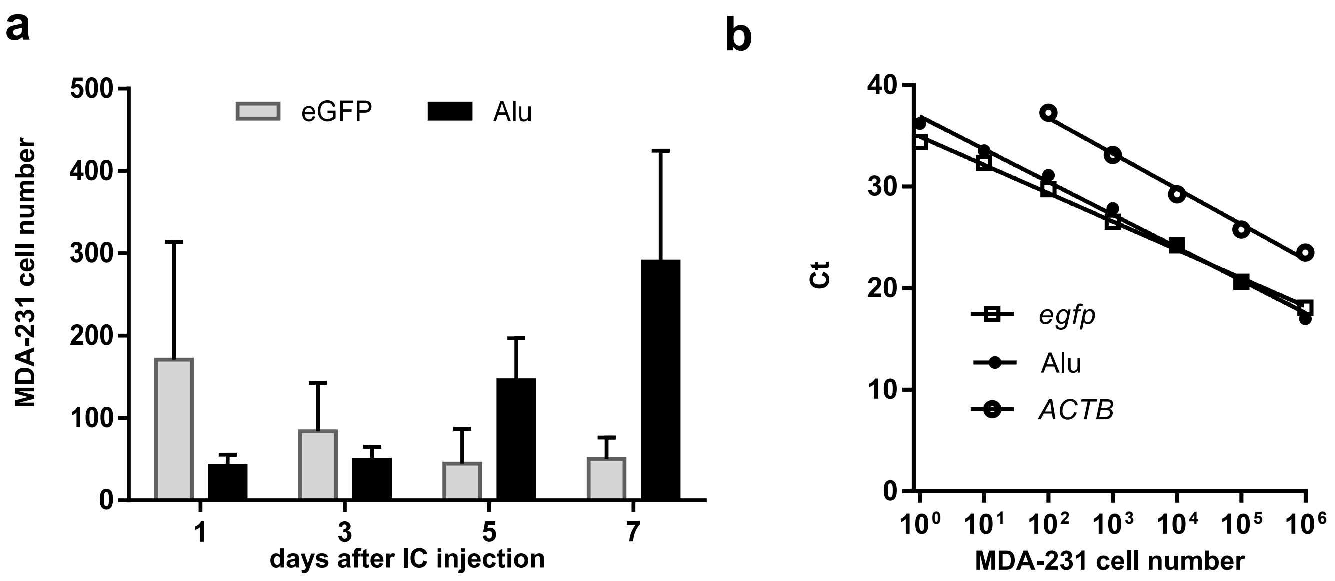

Supplementary Figure 3. eGFP mRNA expression can be used to quantify cancer cell number. (a) In vivo comparison of eGFP and Alu qPCR quantification of metastatic tumor cell number in femora of mice after intracardiac injection (n=5 mice at each time point). (b) In vitro comparison ct values from qPCR of *egfp*, Alu, and *ACTB* after Trizol RNA and DNA extraction of differing numbers of MDA-MB-231VU cells (n=3).

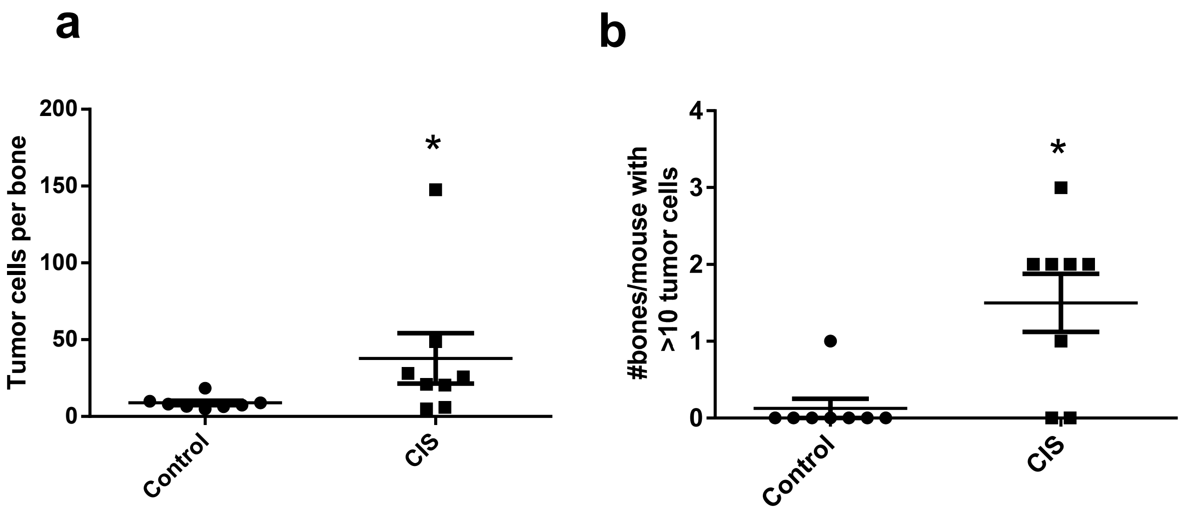

Supplementary Figure 4. Alu qPCR can quantify changes in bone metastatic cell number in a murine stress model. mRNA expression of IL-6 according to number of tumor cells (a) in bones harvested at 3-7 days after intracardiac injection of MDA-231VU with MDA-231VU cells detected by ALU qPCR 5 days after intracardiac injection. Number of MDA-231VU cells detected in femora (b) of control mice vs. chronically immobilized stress treated mice (CIS) (n=8, p<0.005, t-test with Welch's correction) . Number of bones with more than 10 detected cells per mouse, d3 (n=8, p<0.005, Fisher's exact test).
